# New Chemometrics Mode Based on Adjacent Data Points’ Differences for the Simultaneous Determination of Clopidogrel, Atorvastatin, and Aspirin in their Combined Ternary Drug Formulation

**DOI:** 10.3797/scipharm.1401-21

**Published:** 2014-03-24

**Authors:** R’afat Mahmoud Nejem, Mahmoud Mohamed Issa, Alaa Abu Shanab, Raluca-Ioana Stefan-Van Staden, Hassan Y. Aboul-Enein

**Affiliations:** ^1^ Analytical Chemistry, Department of Chemistry, Alaqsa University, P.O.Box 4051, Gaza, Palestine.; ^2^ Pharmaceutical Analytical Chemistry, Department of Chemistry, Alaqsa University, P.O.Box 4051, Gaza, Palestine.; ^3^ Inorganic Analytical Chemistry, Department of Chemistry, Alaqsa University, P.O.Box 4051 Gaza, Palestine.; ^4^ National Institute of Research for Electrochemistry and Condensed Matter, Bucharest, Romania.; ^5^ Pharmaceutical and Medicinal Chemistry Department, Pharmaceutical and Drug Industries Research Division, National Research Center, Dokki, Cairo 12311, Egypt.

**Keywords:** Aspirin, Clopidogrel bisulphate, Atorvastatin calcium, DBADP, MCR

## Abstract

A new method is proposed for the analysis of a ternary mixture composed of clopidogrel, atorvastatin, and aspirin without prior separation steps. The method combines the advantages of the mean centering of ratio spectra and derivative spectrophotometric methods. It is based on using the difference between adjacent data points in the absorbance spectra. The principal advantage of this method is the use of absorbance data, and not derivative data; hence the signal-to-noise ratio is not diminished. The mathematical explanation of the procedure is illustrated. Beer’s law was valid in the concentration range 0.3–35 μg.mL^-1^ for CLOP, 0.5–30 μg.mL^-1^ for ATOR, and 1–40 μg.mL^-1^ for ASP. Mean recoveries were obtained as 100.2, 100.1, and 100.2% for CLOP, ATOR, and ASP, respectively, in the prepared synthetic mixtures. The method has been successfully applied to the simultaneous determination of ternary mixtures of aspirin, clopidogrel bisulphate, and atorvastatin calcium. The analytical characteristics of the method were calculated. The results showed that the new method is simple, rapid, accurate, and precise.

## Introduction

Aspirin (ASP) is often used as an analgesic, antipyretic, anti-inflammatory, and anti-platelet. Clopidogrel bisulphate (CLOP) is an anti-platelet agent. It has been shown to prevent ischemic stroke, myocardial infarction, and vascular disease. Atorvastatin calcium (ATOR) is used for lowering blood cholesterol (Scheme 1).

**Sch. 1.**
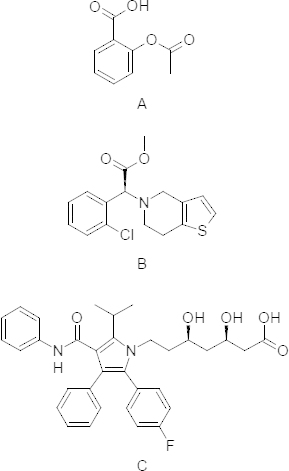
Aspirin (a), Clopidogrel (b), and Atorvastatin (c)

A combination of ASP, CLOP, and ATOR in the form of a capsule formulation is available on the market. Clinical trials showed that combination therapy, when used in dyslipidemic patients with coronary heart disease, reduced cardiovascular events. Consequently, the analysis of a ternary mixture of ASP, CLOP, and ATOR is very important.

The official monographs describe the procedure for the individual assay of ASP [1, 2], CLOP [1], and ATOR [2–4]. The literature survey revealed that very few analytical methods, such as spectrophotometry [5], HPTLC [6], and HPLC [7, 8], have been reported for the simultaneous determination of ASP, CLOP, and ATOR in the ternary mixture. However, the liquid chromatography methods suffered from the extraction procedure, extensive use of expensive solvents, and from being time-consuming. On the other hand, the multivariate calibration procedures are mathematically complex, so much so, that some analysts view them as black boxes and tend to avoid them [9]. The main disadvantage of derivative spectrophotometry is its low reproducibility [10]. So the application of this technique required careful selection of the mathematical parameters. The double divisor-ratio spectra derivative method [11–13] also cannot be popularized, because it can only be used for the mixtures that the ratio of the concentrations of two interfering compounds is known.

Recently, Afkhami and Bahram proposed a new mean centering of ratio spectra method for the simultaneous determination of ternary mixtures [14, 15]. They found that the analytical characteristics obtained by the mean centering of ratio spectra method are significantly better than those obtained by derivative one.

In this paper, a new and simple method was developed for the simultaneous determination of a ternary mixture, without prior separation steps. This method is based on the use of the difference between adjacent data points in the absorbance spectra. There is a mathematical difference between the derivative and adjacent difference methods. The adjacent difference method combines the advantages of the mean centering of ratio spectra and derivative methods. The use of the adjacent difference method offered some advantages over the derivative method:

1. Derivative determines several components if and only if the measurement’s height of the derivative peak of the analyte is performed at those wavelengths, at which the spectra of other components undergo zeroing.
2. Adjacent difference method permits the determination of each compound without interference from other compounds.
3. Derivative gives a broad band, but the adjacent difference has a signal (very sharp band).
4. The advantage of the derivative spectra is at least partially offset by the degradation in the signal-to-noise ratio that accompanies obtaining derivatives.
5. Adjacent difference method eliminates the derivative steps and therefore the signal-to-noise ratio is enhanced.

The method has been applied successfully for the simultaneous determination of ASP, CLOP, and ATOR in their ternary mixture. Furthermore, the mean centering of the ratio spectra as an alternative method has been applied for the resolution of the above-mentioned ternary mixture.

## Theoretical Background

To explain the difference between the adjacent data point expression, let us consider a five-dimensional vector (V):


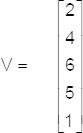


The difference between the adjacent data point (D) of this column can be written:


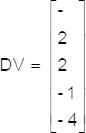


In particular, rules for differentiating between the adjacent data points may be stated as follows:


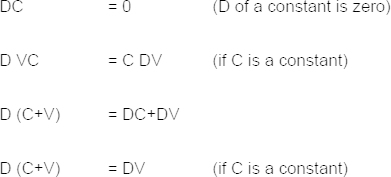


The absorbance for a ternary mixture (ASP, ATOR, and CLOP) is the sum of the absorbance obtained for each individual, then:


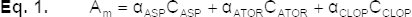


where A_m_ is the vector of the absorbance of the mixture, α_ASP_, α_ATOR_, and α_CLOP_ are the absorptivity vectors of ASP, ATOR, and CLOP, and C_ASP_, C_ATOR_, C_CLOP_, are the concentrations of ASP, ATOR, and CLOP, respectively.

For a ternary mixture of ASP, ATOR, and CLOP, if equation (1) is divided by α_ATOR_ corresponding to a spectrum of a standard solution of ATOR in a ternary mixture, the first ratio spectra is obtained in the form of equation (2):


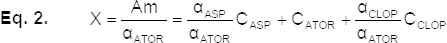


The difference between the adjacent data point of equation (2) is:


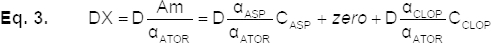


By dividing equation (3) by 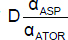, the second ratio spectra is obtained as equation (4):


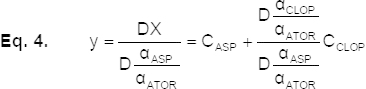


and then:


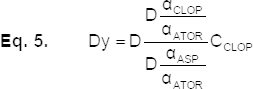


Finally:


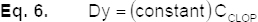


Equation (6) is the mathematical explanation of the new method, which permits the determination of the concentration of each compound in the solution without interference from the other compounds of the ternary mixture. As equation (6) shows, there is a linear relationship between the amount of Dy and the concentration of CLOP in the solution.

A calibration curve could be constructed by plotting Dy against different concentrations of CLOP in the standard solution of CLOP or in the standard ternary mixtures. For more sensitivity, the amount of Dy corresponding to a maximum or minimum point could be measured. Calibration graphs for ASP and ATOR could also be constructed as described for CLOP.

## Experimental

### Apparatus

A Shimadzu (Kyoto, Japan) UV-1650 PC, UV-Visible double-beam spectrophotometer with two matched 1-cm path-length quartz cells was used. The subsequent statistical manipulations were performed by transferring the spectral data to the Microsoft Excel 2010 program and processing them with the standard curve fit package and matrix calculation.

### Reagents

Double distilled water and analytical reagent grade chemicals were used. A stock solution of atorvastatin (1000 μg.mL^-1^) was prepared by dissolving 0.11 g of atorvastatin calcium in 100 ml methanol (Merck). A stock solution of clopidogrel was prepared by dissolving 0.1333 g of clopidogrel bisulphate in100 ml methanol. A standard solution of aspirin (1000 μg.mL^-1^) was prepared by dissolving 0.10 g of aspirin in 100 ml methanol.

Aspirin, atorvastatin calcium, and clopidogrel bisulphate were kindly donated by Middle East Pharm. The commercial product ASPTORGREL capsules (produced by Middle East Pharm, Palestine, Batch no 39712 containing 75 mg of aspirin, 10 mg atorvastatin, and 75 mg of clopidogrel per capsule) was analyzed.

### Procedure

#### Difference Between Adjacent Data Point Method (DBADP)

A calibration graph for CLOP was obtained by recording and storing the spectra of standard solutions containing different concentrations of CLOP, ATOR, and ASP. The stored spectra of the CLOP solutions were divided by the standard spectra of ATOR according to equation (2). Then, the differentiations between the adjacent data points of these vectors with respect to wavelength were obtained according to equation (3). After that, the residual vector was divided by 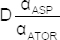 according to equation (4). The minimum or maximum of the differentiations between the adjacent data points of the latter vectors with respect to wavelength was used for the construction of the calibration curve for CLOP. For the prediction of the concentration of CLOP in the synthetic ternary mixtures and real samples, the same procedure was used, expecting that the spectra of the mixture were used instead of the spectra of the standard solution of CLOP.

The construction of the calibration curves for the other active compounds and also their prediction steps was performed as described for CLOP.

#### Mean Centering of Ratio Spectra Method (MCR)

The developed MCR method depended on the mean centering of ratio spectra and has been applied for resolving binary and ternary mixtures in complex samples with unknown matrices. The mathematical explanation of the method was illustrated by Afkhami and Bahram [14, 15].

## Results and Discussion

**Fig. 1.**
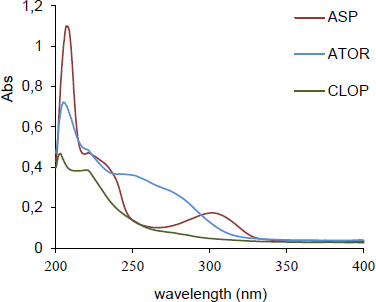
The zero-order spectra (5 μg.mL^-1^) of (1) CLOP; (2) ATOR; (3) ASP in methanol

As Fig. 1 shows, the absorption spectra of CLOP, ATOR, and ASP in methanol overlapped in the region 200–340 nm. The focus of this work was to develop a new method to resolve this overlapping and furthermore, to develop the MCR method for the resolution of these drugs in their ternary mixtures.

### Difference Between Adjacent Data Point Method (DBADP)

The absorption spectra of the prepared solution 1–35 μg.mL^-1^ CLOP, 0.5–30 μg.mL^-1^ ATOR, and 1–40 μg.mL^-1^ ASP were measured in the range of 230–310 nm.

For CLOP, the recorded spectra were divided by the standard spectrum of 1 μg.mL^-1^ ATOR to obtain the first ratio spectra and therefore, the differences between the adjacent data points of the first ratio spectra were obtained. These vectors were then divided by 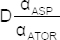 and in the same way, the second ratio spectra could be obtained.

For ASP, the recorded spectra were divided by the standard spectrum of 1 μg.mL^-1^ ATOR to obtain the first ratio spectra and after dividing by 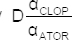, the second ratio spectra of ASP could be obtained.

For ATOR, the recorded spectra were divided by the standard spectrum of 1 μg.mL^-1^ CLOP to obtain the first ratio spectra and after dividing by 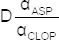, the second ratio spectra of ATOR could be obtained.

**Fig. 2.**
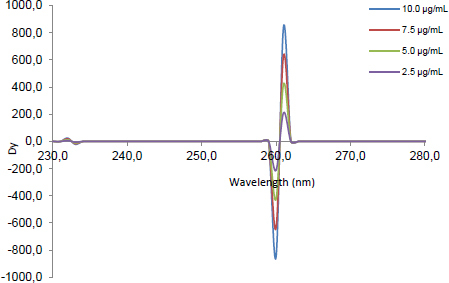
The second ratio spectra of different concentrations (2.5, 5, 7.5, and 10 μg.mL^-1^) of CLOP using the DBADP method

**Fig. 3.**
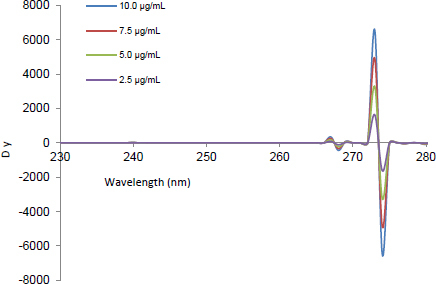
The second ratio spectra of different concentrations (2.5, 5, 7.5, and 10 μg.mL^-1^) of ASP using the DBADP method

**Fig. 4.**
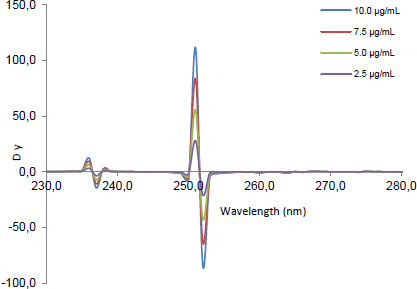
The second ratio spectra of different concentrations (2.5, 5, 7.5, and 10 μg.mL^-1^) of ATOR using the DBADP method

The signals of the second ratio spectra at 260 (Fig. 2), 273 (Fig. 3), and 252 nm (Fig. 4) for CLOP, ASP, and ATOR, respectively, were measured and plotted against the corresponding concentrations of each drug to construct their calibration curves. The spectra of the different synthetic mixtures containing different ratios of CLOP, ASP, and ATOR were recorded and the previously explained procedure was performed to predict the concentration of each compound in the mixture.

In order to optimize the new method, the effect of the divisor on the selectivity of the methods has been tested. Different concentrations of CLOP, ASP, and ATOR were tested. It was found that the divisor had a great effect on the selectivity of the determination of the drugs, where reproducible and good results have been obtained upon using ATOR (for CLOP and ASP) and CLOP (for ATOR) as divisors. On the other hand, changing the concentration of the divisor had a great effect on the slope, intercept, and correlation coefficient of the calibration equations. Therefore, 1 μg.mL^-1^ for each drug was used as the divisors (Table 1). The amount of Δλ had no effect on the signal of the second ratio spectra. A Δλ of 1.0 nm was used.

**Tab. 1.** Analytical characteristics for the analysis of CLOP, ASP, and ATOR by the DBADP method

Drug	λ	Calibration equations (Y =…)	SE^a^	R^2^	Linear Range (μg.mL^-1^)	LOD (μg.mL^-1^)	Intraday (RSD)	Interday (RSD)
CLOP	260.0	-84.10X – 7.30	0.19	0.9994	0.30–35.0	0.08	0.88	0.83
ASP	273.0	648.5X + 25.3	0.18	0.9991	0.10–40.0	0.02	0.58	0.67
ATOR	252.0	10.86X + 3.36	0.10	0.9998	0.50–30.0	0.15	0.80	0.92

^a^ 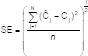

In the method, the parameters used and the calibration data are shown in Table 1. Beer’s law was valid in the concentration range 0.3–35 μg.mL^-1^ for CLOP, 0.5–30 μg.mL^-1^ ATOR, and 1–40 μg.mL^-1^ ASP. Mean recoveries and relative standard deviations (R.S.D) (n=5) of the method were obtained as 100.2 and 1.34% for CLOP, 100.1% and 1.51% ATOR, and 100.2 and 0.96% ASP, respectively, in the synthetic mixtures prepared as shown in Table 2. The repeatability of the method was determined by analyzing the capsules at different time intervals on the same day and on three different days. Results of the precision studies for intraday and interday are shown in Table 1. The limit of detection (defined as the concentration equivalent to three times the standard deviation of five replicate measurements of the blank) and standard error are also shown in Table 1.

The prediction error of a single component in the mixtures was calculated as the relative standard error (R.S.E.) of the prediction concentration [16].


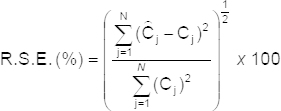


where N is the number of samples, C_j_ is the concentration of the component in the mixture, and 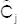 is the estimated concentration. The total prediction error of N samples is calculated as follows:


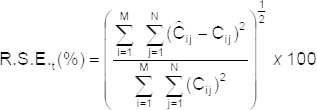


where C_ij_ is the concentration of the component in the j^th^ sample and 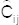 is its estimation. Table 2 also shows the single and total relative errors for the ternary mixtures.

**Tab. 2.** Results of several synthetic mixtures for the analysis of CLOP, ASP, and ATOR by the DBADP method

Taken (μg.mL^-^)			Found (μg.mL^-^)			Recovery (%)		
CLOP	ASP	ATOR	CLOP	ASP	ATOR	CLOP	ASP	ATOR
2.50	2.50	2.50	2.54	2.46	2.43	101.60	98.40	97.20
5.00	5.00	5.00	4.92	5.08	5.09	98.40	101.60	101.80
10.00	10.00	10.00	10.00	9.81	9.79	100.00	98.10	97.90
15.00	15.00	15.00	14.60	14.80	14.90	97.30	98.60	99.30
7.50	7.50	1.00	7.57	7.65	0.97	100.90	102.00	97.00
15.00	15.00	2.00	14.87	15.23	2.04	99.10	101.50	102.00
22.50	22.50	3.00	22.16	22.70	2.91	100.70	102.00	97.00
30.00	30.00	4.00	30.09	30.16	4.09	100.30	100.20	102.20
	Mean recovery					99.80	100.30	99.30
	(±SD)					±1.33	±1.70	±2.55
	R.S.E_t_ (100%)					1.42	1.02	1.49
	R.S.E_t_(100%)						1.34	

### Mean Centering of Ratio Spectra Method (MCR)

The absorption spectra for each drug were measured in the range of 200–340 nm. For CLOP, the recorded spectra were divided by the standard spectrum of 1.0 μg.mL^-1^ ATOR to obtain the first ratio spectra which was then mean-centered. These vectors were then divided by the mean center of 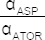 and therefore, the mean centering of the second ratio spectra were obtained. In the same way, the second ratio spectra of ASP and CLOP could be obtained.

**Fig. 5.**
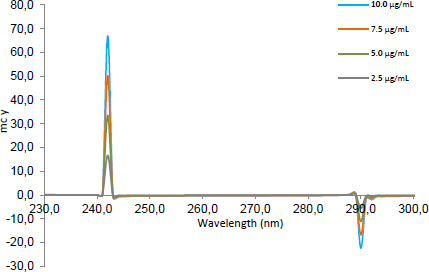
The second ratio spectra of different concentrations (2.5, 5, 7.5, and 10 μg.mL^-1^) of CLOP using the MCR method

**Fig. 6.**
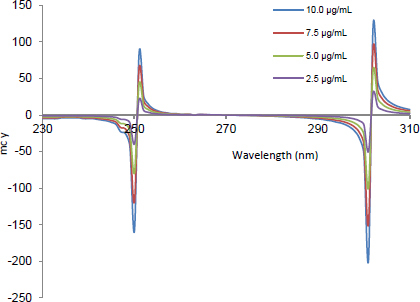
The second ratio spectra of different concentrations (2.5, 5, 7.5, and 10 μg.mL^-1^) of ASP using the MCR method

**Fig. 7.**
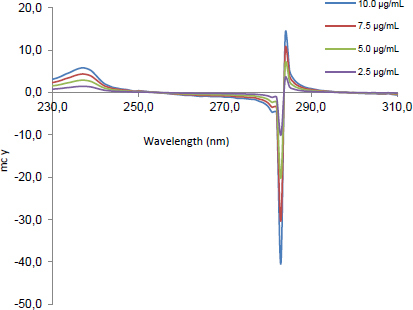
The second ratio spectra of different concentrations (2.5, 5, 7.5, and 10 μg.mL^-1^) of ATOR using the MCR method

The mean centered values of the second ratio spectra at 242 (Fig 5), 301 Fig (6), and 283 nm (Fig 7) for CLOP, ASP, and ATOR, respectively, were measured and plotted against the corresponding concentrations of each drug to construct their calibration curves.

The spectra of the different synthetic mixtures and real samples were recorded and the MCR method was performed to predict the concentration of each compound in the mixture. Table 3 shows the linear regression parameters for the calibration data for the simultaneous determination of CLOP, ASP, and ATOR in their ternary mixture. Relative standard deviations, mean recoveries, and relative standard error are given in Table 4.

**Tab. 3.** Analytical characteristics for analysis of CLOP, ASP, and ATOR by the MCR method

Drug	λ	Calibration equations	R^2^	Linear Range (μg.mL^-1^)	LOD (μg.mL^-1^)
CLOP	242.0	y = 6.91X - 2.27	0.9990	1.00-35.00	0.10
ASP	301.0	y = -19.1X + 8.55	0.9989	0.10-40.00	0.08
ATOR	283.0	y = -4.40X - 3.61	0.9992	0.50-30.00	0.20

Good concordance was observed for the results of Table 2 and Table 4 by the application of the two methods described in this paper.

**Tab. 4.** Results of several synthetic mixtures for analysis of CLOP, ASP, and ATOR by the MCR method

Taken (μg.mL^-^)			Found (μg.mL^-^)			Recovery (%)		
CLOP	ASP	ATOR	CLOP	ASP	ATOR	CLOP	ASP	ATOR
2.50	2.50	2.50	2.41	2.43	2.57	96.40	97.20	102.8
5.00	5.00	5.00	5.05	5.09	5.06	101.00	101.8	101.2
10.0	10.0	10.00	10.06	10.21	10.11	100.60	102.1	101.1
15.0	15.0	15.00	15.4	15.10	15.20	102.60	100.6	101.3
7.50	7.50	1.00	7.64	7.66	1.00	101.80	102.1	100.0
15.0	15.0	2.00	15.20	15.30	1.95	101.30	102.0	97.50
22.5	22.5	3.00	22.32	22.89	2.92	99.20	101.7	97.60
30.0	30.0	4.00	29.21	29.63	3.91	97.30	98.70	97.70
	Mean recovery					100.0	100.8	99.9
	R.S.E_t_ (100 %)					2.09	1.52	1.42
	R.S.E_t_ (100 %)						1.77	

### Application

To evaluate the applicability of the proposed DBADP and MCR methods, both methods were applied to the simultaneous determination of CLOP, ASP, and ATOR in commercially available capsules. Five replicate determinations were made. Satisfactory results were obtained for each drug (Table 5). The results of the new DBADP methods were compared with those of the MCR method. Statistical comparison between the results was performed with regards to accuracy and precision using the t-test and F-ratio at a 95% confidence limit. There was no significant difference between the results.

**Tab. 5.** Determination of CLOP, ASP, and ATOR in commercial Asptorgrel capsules using the proposed method

Sample No.	DBADP (mg.kg^-1^)			MCR (mg.kg^-1^)		
	CLOP	ASP	ATOR	CLOP	ASP	ATOR
1	73.65	75.37	10.11	75.60	74.32	9.956
2	75.60	75.67	10.23	75.82	74.10	10.09
3	74.70	75.82	99.61	73.42	74.70	10.06
4	75.97	74.92	98.94	73.80	73.12	10.08
5	76.05	74.02	9.88	74.32	76.12	9.92
Mean	75.19	75.16	10.01	74.59	74.47	10.02
±S.D	±1.01	±0.72	±0.156	±1.07	±1.09	±0.08
*F*^a^	0.891	0.436	3.51			
*t*^b^	0.803	0.968	0.100			

^a^ *F*_0.05, 4.4_ = 6.3;

^b^ t_0.05,8_ = 2.306.

## Conclusion

A comparative study of the use of the DBADP and MCR methods for the resolution of a ternary drug mixture of CLOP, ASP, and ATOR has been accomplished showing that the two methods provide a clear example of the high resolving power of these techniques. These methods have the advantages of high sensitivity, extremely low detection limit, rapid analysis, and inexpensive instruments. In contrast to the derivative, our new DBADP method depends on absorbance data, not derivative data; hence the signal-to-noise ratio is not diminished. Our new DBADP method combines the advantages of the mean centering of ratio spectra method with those of derivative spectrophotometry.
